# Fecal microbiota and metabolites in the pathogenesis and precision medicine for inflammatory bowel disease

**DOI:** 10.1093/pcmedi/pbae023

**Published:** 2024-09-23

**Authors:** Long Ju, Zhimin Suo, Jian Lin, Zhanju Liu

**Affiliations:** Center for Inflammatory Bowel Disease Research and Department of Gastroenterology, Shanghai Tenth People's Hospital of Tongji University, Shanghai 200072, China; Department of Gastroenterology, Huaihe Hospital of Henan University, Kaifeng 475000, China; Center for Inflammatory Bowel Disease Research and Department of Gastroenterology, Shanghai Tenth People's Hospital of Tongji University, Shanghai 200072, China; Department of Gastroenterology, Affiliated Hospital of Putian University, Putian 351100, China; Center for Inflammatory Bowel Disease Research and Department of Gastroenterology, Shanghai Tenth People's Hospital of Tongji University, Shanghai 200072, China

**Keywords:** inflammatory bowel disease, fecal microbiota, metabolite, diagnosis, treatment, pathogenesis

## Abstract

Inflammatory bowel disease (IBD) is a chronic inflammatory disorder of the gastrointestinal tract, and its pathogenesis is believed to be associated with an imbalance between commensal organisms and the intestinal immune system. This imbalance is significantly influenced by the intestinal microbiota and metabolites and plays a critical role in maintaining intestinal mucosal homeostasis. However, disturbances in the intestinal microbiota cause dysregulated immune responses and consequently induce intestinal inflammation. Recent studies have illustrated the roles of the intestinal microbiota in the pathogenesis of IBD and underscored the potential of precision diagnosis and therapy. This work summarises recent progress in this field and particularly focuses on the application of the intestinal microbiota and metabolites in the precision diagnosis, prognosis assessment, treatment effectiveness evaluation, and therapeutic management of IBD.

## Introduction

Inflammatory bowel disease (IBD) is a chronic nonspecific inflammatory disease of the gastrointestinal tract that mainly includes Crohn's disease (CD) and ulcerative colitis (UC). Despite extensive study, the precise etiologies and pathologies of IBD, which are considered to be involved in a complex interplay of genetic predispositions, microbial alternations, dysregulated immune responses, and environmental factors, remain elusive [1]. Essential in the pathogenesis of IBD is the disruption of intestinal immune homeostasis, influenced by predisposing factors including the gut microbiota, diet, medications, and psychological stress [2, 3].

The gut microbiota, a key player in this ecosystem, performs critical functions such as nutrient metabolism, maintenance of mucosal barrier integrity, immune modulation, and pathogen defense. The delicate balance between the gut microbiota and metabolites and the intestinal immune system is crucial for intestinal health [4]. Disturbances in this balance have been reported to be linked to various gastrointestinal disorders, including IBD [5], indicating that the gut microbiota diversity and abundances are vital for maintaining intestinal homeostasis [6].

Emerging evidence underscores the pivotal role of the gut microbiota in the onset of IBD and its progression. This review discusses the impacts of the intestinal microbiota on the pathogenesis of IBD and explores their potential in refining the precision diagnosis and treatment of this complex disease.

## Regulation of intestinal microbiota in intestinal mucosal homeostasis

### Physiological functions of the intestinal microbiota

The intestinal microbiota, comprising bacteria, viruses, fungi, parasites, and archaea [7], encompasses >50 bacterial groups with ∼1100 species. The collective mass of the intestinal microbiota is ∼0.2 kg, making up 60% of stool's dry mass [8]. Predominantly, *Firmicutes, Bacteroidetes*, and *Actinobacteria* constitute 90% of the gut microbiota, with *Proteobacteria* and *Fusobacteria* being less common [6]. Notably, *Firmicutes*, known for their production of butyric acid, a type of short-chain fatty acid (SCFA), play a crucial role in sustaining the health of the intestinal epithelia [9].

The intestinal microbiota performs vital physiological functions that contribute to the host's overall well-being, including assisting in food digestion and energy production, regulating immune responses, protecting the intestinal lining, regulating fat storage, stimulating intestinal angiogenesis [9], producing a variety of growth factors, shielding against pathogen invasion, and ensuring the intestinal microecological balance [7]. For example, *Bifidobacterium longum* has the capacity to balance the intestinal flora, mitigate intestinal inflammation by reducing proinflammatory cytokines and reactive oxygen species, and fortify the intestinal epithelial barrier integrity [10], which is suggestive of its therapeutic potential in IBD management.

The intestinal microbiota also provides important metabolites such as SCFAs, indoles, and bile acids [11, 12], and produces SCFAs, mainly including acetate, propionate, and butyrate. *Bacteroidetes* mainly produce acetate and propionate, while *Firmicutes* mainly produce butyrate. SCFAs enhance the barrier function of intestinal epithelial cells and also have immunomodulatory effects, such as reducing pro-inflammatory cytokines and stimulating the generation of regulatory T cells (Tregs). The intestinal microbiota converts tryptophan into a range of indole metabolites, and indoles play a regulatory role in intestinal mucosal immunity [13]. Additionally, primary bile acids are processed by the intestinal microbiota into secondary bile acids, which play an important role in preventing bacterial overgrowth, modulating mucosal immunity, and protecting intestinal epithelial integrity [7].

### Intestinal microbiota antigens maintain intestinal mucosal immune homeostasis

Intestinal microbiota-derived antigens play dichotomous roles in maintaining intestinal mucosal homeostasis by either boosting mucosal immune responses or fostering immune tolerance. Increasing lines of evidence have demonstrated that certain microbial antigens can escalate mucosal immune responses, thus inducing CD4^+^ T cell activation and proliferation in the gut mucosa and facilitating them to secrete proinflammatory cytokines [14]. This event results in intestinal mucosal inflammation and tissue damage. A notable example involves the *segmented filamentous bacteria* (SFB), known for their capacity to enhance the generation of T helper 17 (Th17) cells in the small intestinal lamina propria, a key area for mucosal defense and autoimmune disease development [15]. Studies using fecal microbiota transplantation (FMT) from SFB-infected mice into germ-free or normal mice have shown a significant Th17 cell accumulation, highlighting the potential role of SFB in the induction of inflammatory processes in gut mucosa [15, 16].

Conversely, other evidence has also shown that several microbiota components modulate immune responses towards immune tolerance, thus aiding in inflammation control. *Bacteroides fragilis*, for example, leverages its polysaccharide A antigen to mitigate experimental colitis in mice by reducing proinflammatory interleukin (IL)-17A production, a process dependent on IL-10 production from CD4^+^ T cells [17]. In addition, *Enterococcus faecium* could activate the nucleotide-binding oligomerization domain-containing protein 2 (NOD2) gene in myeloid cells, an important gene involved in the regulation of the inflammatory response to bacterial antigens [18], thereby upregulating IL-1β, which subsequently enhances the secretion of IL-22 by lymphoid cells and exerts anti-inflammatory effects [19]. Moreover, the intestinal Tregs, crucial for immunoprotection, are modulated by the intestinal microbiota. Previous studies have also proven that specific strains of *bifidobacteria, lactobacilli*, and *B. fragilis* as well as particular *Clostridium* clusters, could enhance Treg accumulation in gut mucosa, thus providing a promising approach in the prevention of colitis and other inflammatory conditions [20,21].

### Intestinal microbial metabolites participate in intestinal mucosal homeostasis

The gastrointestinal tract serves as a complex ecosystem where a diverse community of microorganisms produces a variety of metabolites with significant impacts on host physiology [13]. These metabolites, particularly SCFAs like acetate, propionate, and butyrate, are pivotal in maintaining intestinal mucosal homeostasis. Accumulating lines of evidence have highlighted that SCFA levels are notably reduced in IBD patients and murine colitis models, whereas SCFA supplementation mitigates colitis symptoms [22]. SCFAs are instrumental in preserving intestinal health, providing energy to the colonic cells, and modulating immune responses to reduce intestinal mucosal inflammation [23], and play a key role in energy metabolism regulation. Acetate influences satiety and fat storage, and propionate affects intestinal motility and energy metabolism via glucagon-like peptide 1 (GLP1) secretion. Moreover, butyrate, as the primary energy source for colonic epithelial cells, enhances GLP1 production [13]. Beyond energy metabolism, SCFAs have profound effects on the intestinal immune system, fostering the development of B cells and Tregs, contributing to the intestinal mucosal integrity, and possessing anti-inflammatory properties through the production of IL-18 and inhibition of histone deacetylases [13]. Additionally, SCFAs regulate intestinal IgA production, essential for maintaining gut homeostasis and combating intestinal inflammation. This regulation is driven by acetate that acts through G protein-coupled receptor 43 (GPR43) to prompt dendritic cells to convert vitamin A into retinoic acid, thereby stimulating IgA production by B cells [24]. Furthermore, SCFAs influence cellular immunity by Treg cell differentiation, which further facilitates IL-10 and IL-22 production, essential for maintaining intestinal balance and controlling gut inflammation [25]. Specifically, butyrate has been shown to promote the differentiation of CD4^+^ T cells into Tregs, thus contributing to potential therapeutic benefits to IBD patients [26]. In addition, SCFAs also constrain the production of pro-inflammatory cytokines by neutrophils, thereby alleviating mucosal inflammatory damage [27].

Bile acids are derivatives of cholesterol, and the intestinal microbiota markedly influences the synthesis and metabolism of bile acids [28]. The primary bile acids are converted into secondary bile acids by the intestinal microbiota, thus greatly expanding the molecular diversity in the intestinal environment. Previous study has demonstrated that *B. longum* CECT 7894, which contains bile salt hydrolase genes, improves the efficacy of anti-TNF monoclonal antibody [i.e. infliximab (IFX)] in a murine colitis model through the regulation of the metabolism of bile acids [29]. Several therapeutic approaches, such as diet, probiotics, FMT, and ursodeoxycholic acid, could alleviate intestinal mucosal inflammation by regulating bile acids and the intestinal microbiota [30]. Evidence has shown that bile acids play an important role in the maintenance of intestinal microbiota homeostasis and the delicate balance of the mucosal immune system, and generally possess an anti-inflammatory effect. They inhibit the assembly of inflammatory vesicles, decrease the expression of proinflammatory cytokines in macrophages and dendritic cells, and prevent the proinflammatory capacity of monocytes. Moreover, bile acids constrain the differentiation and function of Th17 cells [31], and upregulate the differentiation of Tregs, downregulate proinflammatory cytokines such as IL-17A/F and TNF-α, and upregulate anti-inflammatory cytokines such as IL-10 and transforming growth factor-β (TGF-β). Additionally, bile acids also protect intestinal barrier integrity by enhancing tight junction molecule expression [32].

Tryptophan is an essential aromatic amino acid that humans must obtain from their diet. Poultry, fish, oats, and dairy products are common sources of dietary tryptophan. Plasma tryptophan is decreased in IBD patients due to dysregulated gut microbiota metabolism [13] and is negatively correlated with IBD disease severity [33]. Tryptophan can be converted into a series of indole metabolites by the intestinal microbiota. Indoles promote the release of GLP1, and indole derivatives act as agonists of the transcription factor aryl hydrocarbon receptor, thereby affecting mucosal immunity and CD4^+^ T cell activation and proliferation [13]. Consistently, supplementation with *Lactobacillus*-producing tryptophan catabolite could reduce susceptibility to inflammatory disease [34].

The intestinal microbiota also produces other metabolites, such as histamine, which plays a role in maintaining immune responses in gut mucosa. Histamine is a biogenic amine found in a wide variety of cells. The main source of histamine is food, but it can also be synthesized and regulated by the intestinal microbiota. Histamine has proinflammatory effects, and is found to be increased in the inflamed mucosa of IBD patients [35]. Moreover, mast cells also produce histamine upon antigen stimulation in the inflamed mucosa of IBD patients, indicating that mast cells are also involved in the pathogenesis [36].

## Dysbiosis of the intestinal microbiota is involved in the development of IBD

### Alterations in the intestinal microbiota in IBD patients

**Figure 1.**
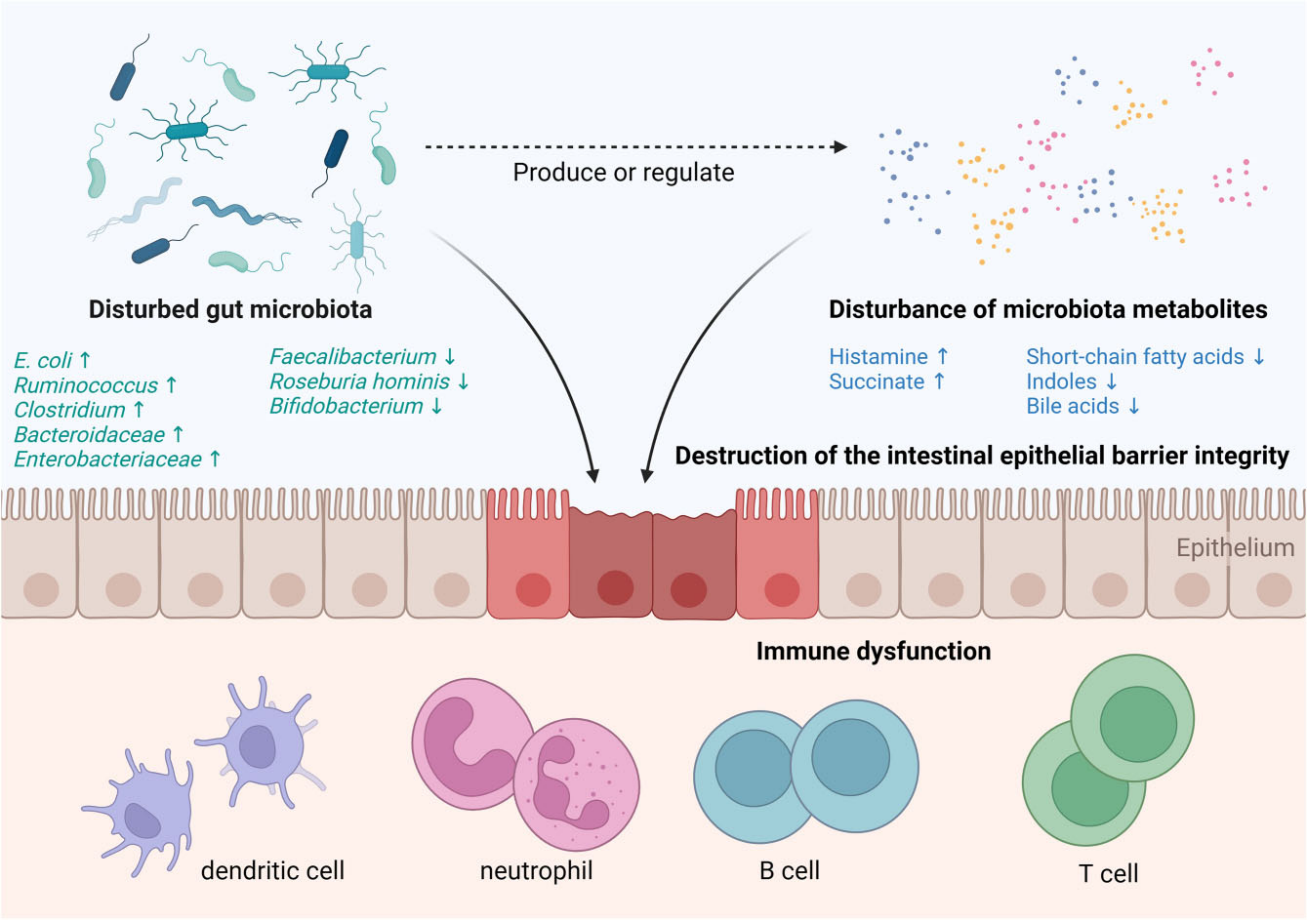
Gut microbiota and metabolites are involved in the pathogenesis of IBD. Gut microbiota and metabolite disturbance in IBD includes a decrease in commensal bacteria and anti-inflammatory metabolites such as SCFAs, and an increase in pathogenic bacteria and proinflammatory metabolites such as histamine and succinate, which then induce compromised intestinal barrier integrity and dysregulated immune responses in gut mucosa, contributing to the pathogenesis of IBD.

IBD patients have disrupted intestinal microbiota and metabolites, characterized by reduced bacterial diversity, increased invasive microbiota (such as *Proteus* and *Escherichia coli*) and decreased protective microbiota (such as *Bifidobacterium*). Moreover, the content of SCFAs in the stool of IBD patients is significantly lower than that of healthy donors, which is consistent with the low frequencies of SCFA-producing microbiota (such as *Faecalibacterium* and Methanogenic archaea) in IBD patients [37]. Such alterations are involved in the pathogenesis of IBD [38], causing damage to the intestinal barrier and dysregulated immune response in the gut mucosa [39, 40] (Fig. 1).

In addition to the alterations in intestinal microbiota observed in IBD patients compared to healthy individuals, distinct microbial profiles are associated with each IBD behavior. IBD encompasses two primary subtypes, namely UC and CD, which can be further stratified based on disease location and comorbidities. A recent Mendelian randomization analysis exploring gut microbiota across 10 IBD subtypes revealed that the family *Defluviitaleaceae* is associated with a reduced risk of colonic CD, while the genus *Lachnospiraceae ND3007* group and the genus *Hungatella* are linked to a decreased risk of left-sided UC. Generally, *Hungatella, Acidaminococcaceae*, and 13 other microbial taxa are identified as protective factors for various IBD subtypes, whereas *Terrisporobacter, Anaerostipes*, and 21 other microbial taxa are associated with an increased risk for different IBD subtypes [41]. These findings underscore the potential for modifying gut microbiota composition as a strategy for preventing and improving the prognosis of IBD.

Many other aspects of the altered intestinal microbiota in patients with IBD are also apparant. Increasing lines of evidence have shown that bacterial levels are disturbed in the inflamed mucosa [42], such as an increase in facultative anaerobes such as *E. coli* but a decrease in beneficial obligate anaerobes like *Faecalibacterium prausnitzii* and *Roseburia hominis*. Moreover, higher numbers of *Ruminococcus torques* and *Ruminococcus gnavus* are present in IBD patients, along with increased abundance and activity of certain *Clostridium* species [43]. In addition, a reduction in fumarate and its derivatives in murine colitis models, along with a decrease in fumarate-producing bacteria, suggests a metabolic shift associated with disease status [44]. At the ecological level, some bacterial species in IBD patients have specific co-abundance relationships compared to healthy controls. For example, *E. coli* shows a positive co-abundance relationship with pro-inflammatory bacteria, but a negative co-abundance relationship with anti-inflammatory bacteria [45], suggesting a disturbance of intestinal microbial interactions that plays an important role in the pathogenesis of IBD.

Advancements in 16S rRNA and shotgun metagenomic sequencing have revolutionized our understanding of the intestinal microbiota, offering a high-resolution view that distinguishes IBD patients from healthy individuals. These technologies accurately analyze intestinal microbial composition from stool or mucosal biopsy samples, generate extensive bioinformatics data through gene sequencing [46], and facilitate the exploration of underlying mechanisms by which intestinal microbes contribute to the onset and progression of IBD. Recently, several studies have identified new loci associated with IBD [47–49], paving the way for better understanding of host–microbe abnormalities in IBD [50]. Some antimicrobial genes are observed to be overexpressed in IBD [51], e.g. expression of the antimicrobial gene regenerating family member 3 (REG3) is abnormally increased in IBD patients [19], while GPR65, which has antibacterial effects, is significantly reduced in the inflamed intestinal epithelium [52]. GPR84, which can be stimulated by lipopolysaccharide, is increased in inflamed colon tissue [53]. These studies help to reveal the mechanism of the involvement of intestinal microbiota in the occurrence and development of IBD and provide an avenue for potential therapeutic intervention.

### Dysregulated intestinal microbiota facilitates intestinal inflammation in IBD

Significant alterations in the composition and functionality of the gut microbiota are present in IBD patients. An upsurge in *Bacteroidaceae* [54] and *Enterobacteriaceae* families has been observed in an experimental colitis model in mice, linked with an increase in intestinal formic acid [55]. Such dysbiosis aggravates the inflammatory processes, highlighting a vicious cycle between the intestinal microbiota imbalance and intestinal inflammation.

Further studies have underscored the intricate relationship between microbiota dysfunction and intestinal inflammation. For example, mice deficient in caspase recruitment domain family member 9 (CARD9) exhibit altered tryptophan metabolism by the gut microbiota, leading to a decrease in IL-22 production and a heightened vulnerability to colitis [56], suggesting that the intestinal microbial metabolite activities are crucial for modulating the immune response and maintaining mucosal integrity. Additionally, experiments involving *Lactobacillus* strains capable of metabolizing tryptophan have shown promising results in mitigating intestinal inflammation, thus underscoring the potential therapeutic benefits of modulating the intestinal microbial metabolism [57]. Moreover, the role of specific microbial metabolites, such as butyrate produced by *Clostridium* strains, has been highlighted in controlling intestinal mucosal inflammation. In line with this, limiting the beneficial microbes exacerbates intestinal inflammation, whereas supplementation of the gut with butyrate-producing probiotics has shown efficacy in alleviating colitis symptoms, indicating that strategic manipulation of the gut microbiota to enhance the presence of beneficial metabolite-producing microbes could offer a novel approach to managing IBD [58].

Regulating the intestinal microbiota of IBD patients appears to directly impact the degree of intestinal inflammation. When a large number of bacteria invade the gut mucosa, neutrophils migrate into the intestinal epithelial area to clear bacterial infection. However, dysfunctional regulation of neutrophils in mucosal homeostasis not only promotes susceptibility to intestinal epithelial damage (e.g. cryptitis and crypt abscess), but also affects intestinal inflammation [59]. *Enterobacteriaceae* is found to be increased in the intestines of early CD patients, and elimination of these bacteria from the intestines has been shown to reduce the degree of mucosal inflammation [60]. A decrease in the abundance of intestinal *F. prausnitzii*, an anti-inflammatory bacterium, in patients with CD has been linked to a higher risk of recurrent inflammation [61]. The supernatants of *F. prausnitzii* have been observed to downregulate nuclear factor (NF)-κB activity [62], and stimulation of peripheral blood mononuclear cells by *F. prausnitzii in vitro* significantly reduces the production of IL-12 and interferon-γ, but facilitates IL-10 production [61]. Notably, oral administration of *F. prausnitzii* or its supernatants to mice significantly reduces the severity of colitis and tends to correct colitis-related dysbiosis. Moreover, a previous study has shown that the supernatant of *F. prausnitzii* culture also produces a microbial anti-inflammatory molecule (MAM), which is a seven peptide anti-inflammatory that significantly reduces the activation of the NF-κB pathway. Consistently, the delivery of the MAM plasmid by *Lactobacillus* has been demonstrated to markedly reduce experimental intestinal inflammation in mice [62]. These data indicate *F. prausnitzii* as a potential treatment option in IBD.

### Dysbiosis of the intestinal microbiota contributes to disease progression by promoting creeping fat formation in CD patients

A previous study highlighted an intriguing aspect of CD pathology: the formation of creeping fat in the mesenteric adipose tissue (MAT) surrounding the intestine [63]. This phenomenon is closely linked to the altered interactions between the host and its intestinal microbiota, along with defects in the intestinal epithelial barrier functions, which facilitates the translocation of the gut microbes into MAT [64]. The mesentery, which connects the intestine to the abdominal wall and supports its blood and lymphatic supply, becomes encased in the creeping fat. Initially, this fat hypertrophy plays a protective role in alleviating intestinal damage and preventing the systemic dissemination of bacterial antigens [65]. However, the dual nature of the creeping fat in CD patients presents a complex scenario. It could mitigate the spread of inflammation and bacteria initially, whereas prolonged accumulation of the creeping fat detrimentally affects the intestinal wall and nearby normal tissues [65]. Accordingly, the adipose tissue in CD patients is not just a passive fat deposit but is actively involved in the immune response, and is enriched with activated immune cells like T cells, B cells, neutrophils, and macrophages. Microbial translocation (e.g. *Clostridium innocuum*) prompts significant pathological changes in the mesentery, leading to tissue remodeling and fibrosis via macrophage activation [65, 66].

This aspect of CD underlines the intricate relationships between the gut microbiota dynamics, immune responses, and the pathological changes in intestinal and adjacent tissues. Understanding the mechanisms underlying creeping fat formation and its effects on disease progression will open new avenues for potential target therapies in CD, focusing on restoring the balance of the intestinal microbiota and reinforcing intestinal barrier functions.

### Oral microbiota translocation and transintestinal migration affect intestinal homeostasis

The intricate connection between the oral microbiota and IBD highlights the complex interplay between different microbial communities within the human body. The oral microbiota, ranking second in abundance only to the gut microbiota, exerts a profound influence on overall health and has been linked to the development of IBD [67]. Alterations in oral microbial composition, often resulting from conditions like periodontitis, have been associated with the pathogenesis of IBD. Studies have shown that periodontitis participates in the pathogenesis of colitis through the swallowing of salivary microbiota [68]. Previous studies have demonstrated that periodontitis exacerbates experimental colitis by introducing oral pathogens, such as *Klebsiella* and *Enterobacteriaceae*, into the gastrointestinal tract, where they incite gut inflammation [69, 70]. Moreover, the migration of Th17 cells, activated in response to oral pathogens, to the gut further underscores the systemic impact of oral microbial imbalances on intestinal health [70]. *Porphyromonas gingivalis*, a notable periodontal pathogen, has been implicated in altering the gut's microbial landscape and immune balance, specifically the Th17/Treg cell ratio, thereby aggravating intestinal inflammation [71].

Several studies have revealed the host–microbe interactions across multiple tissues in CD patients based on spatial omics analysis. Hazardous conditions such as intestinal inflammation, transmural inflammation, and impaired intestinal vascular barrier integrity may promote bacterial dissemination to intestinal and external tissues, accompanied by immune cell activation, and further contribute to the onset and persistence of intestinal inflammation [72, 73]. These data suggest that host microbes not only affect the intestinal mucosa, but also participate in the extraintestinal progression of inflammation by affecting MAT, mesentery, and mesenteric lymph nodes.

The evidence mentioned above underscores the importance of both local and distant microbial communities in the etiology and pathology of IBD, suggesting a broader therapeutic perspective that manages oral health to maintain intestinal homeostasis and prevent disease exacerbation.

## Intestinal microbiota in the precision diagnosis and treatment of IBD

### Intestinal microbiota and metabolites are used for precision diagnosis of IBD

Advances in understanding the intestinal microbiota and metabolites offer new avenues for the precision diagnosis and treatment of IBD. Notably, the genetic signatures of fecal microbes from CD patients are promising as non-invasive biomarkers [74]. One pivotal study analyzed fecal samples from 1418 CD patients and healthy individuals across multiple cohorts and demonstrated the utility of microbial genes in the precision diagnosis, achieving a high accuracy in distinguishing CD from other conditions. Specifically, the phosphotransferase system, particularly the *celB* and *manY* genes, has emerged as a significant contributor to diagnostic performance, thus underscoring the potential of these microbial genes as reliable, non-invasive diagnostic tools for CD [75].

Moreover, analysis of bacterial profiles in fecal samples has shown significant potential for differential diagnosis. A multinational study involving 2045 stool samples identified distinct microbial patterns in CD patients, characterized by reduced diversity and stability. This study identified that eight microorganisms can be used to discriminate CD patients from other diseases. Another study showed that a machine-learning predictive model, developed from fecal microbiota analysis, can effectively diagnose and classify UC and CD [76], highlighting the diagnostic potential of fecal bacterial analysis in clinical practice [77].

Additionally, the discovery of anti-paratuberculosis-nocardia polypeptide antibody as a biomarker opens new doors for assessing CD severity and activity. This antibody, produced in response to *Mycobacterium avium* subsp. *paratuberculosis* infection, has shown excellent predictive value for active CD, further illustrating the burgeoning role of microbial and immune markers in diagnosing and understanding IBD [78].

Bile acids, regulated by the intestinal microbiota, have an excellent diagnostic efficacy in stratifying IBD activity. The ratio of serum primary to secondary bile acids was found to be an ideal index for stratifying IBD activity [79]. These data suggest that microbial metabolites (e.g. bile acids) can be used for the differential diagnosis of IBD.

**Figure 2.**
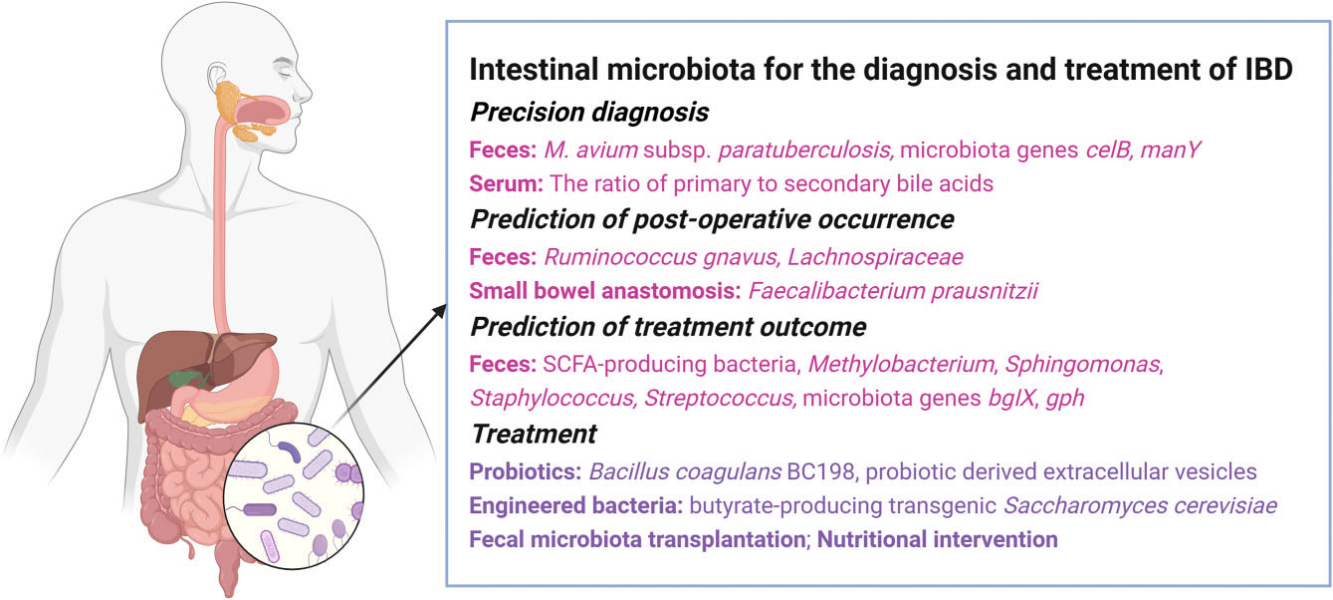
Intestinal microbiota and metabolites have multiple applications in IBD, including precision diagnosis, prediction of post-surgical occurrence and treatment outcome, and effective therapeutic strategies. Feces is a commonly used test sample, and bacteria and bacterial genes in feces are potential biomarkers for IBD.

The heterogeneity of the host genes and gut microbiota in the population leads to the heterogeneity of the host–microbiota interaction. Current progress on host genomics, epigenomics, microbiome, metabolomics, and other omics of IBD, is expected to deepen the understanding of IBD disease characteristics in different cohorts [80] and achieve precision and personalized medicine in IBD. These insights into the microbial and metabolic underpinnings of IBD not only enhance diagnostic accuracy but also pave the way for personalized treatment approaches, leveraging the unique microbial signatures of individuals to tailor interventions (Fig. 2).

### Intestinal microbiota predicts post-operative occurrence of IBD

The predictive power of the intestinal microbiota extends to forecasting surgical outcomes in IBD treatment. For UC patients undergoing colectomy with ileal pouch–anal anastomosis, preoperative fecal bacterial profiles have been linked to postoperative complications like pouchitis. Specific bacterial shifts, an increase in pathogens like *R. gnavus*, and a decrease in beneficial microbes such as those from the *Lachnospiraceae* family, have emerged as potential indicators of post-surgical prognosis, offering a new method to assess the risk of postoperative complications [81].

Moreover, the recurrence of CD after ileocolonic resection can be anticipated by analyzing microbial patterns in the small intestine and at the anastomosis site. Previous studies have identified distinct microbial compositions associated with either recurrence or sustained remission, pointing to the role of specific microbes like *F. prausnitzii* in potentially protecting against mucosal inflammation recurrence [82].

These insights into the diagnostic and prognostic capabilities of the intestinal microbiota underscore their potential as a tool in managing IBD more effectively. By predicting surgical outcomes, clinicians can tailor treatment plans more accurately, enhancing patient care and optimizing treatment efficacy.

### Intestinal microbiota predicts the therapeutic outcome in IBD

The intestinal microbiota and metabolites hold promise in predicting responses to biotherapy for IBD. With fecal microbiota serving as a mirror to the gut's microbial ecosystem, an analysis can inform on the likely success of biological treatments [83].

The characteristics of the fecal microbiota in IBD patients at baseline have the potential to predict multi-level treatment effects [84]. A more diverse intestinal microbiota at baseline may indicate a more abundant anti-inflammatory microbiome, and that microbial diversity at baseline may predict treatment outcomes [85]. A good anti-inflammatory effect is more likely to be achieved when SCFA-producing bacteria are abundant. In addition, microorganisms that cause succinate accumulation are often associated with alleviating intestinal inflammation [86]. Some fecal bacteria and metabolites may predict the outcome of IFX therapy in pediatric CD patients. A multi-omics analysis of stool samples from 49 children with CD and healthy controls showed that the disease severity of CD and the outcome of IFX treatment are correlated with the abundance of certain intestinal bacteria and the levels of metabolites. *Methylobacterium, Sphingomonas, Staphylococcus*, and *Streptococcus* were abundant in the intestines of pediatric CD patients who achieved sustained remission after IFX treatment. At the same time, pediatric CD patients who achieved sustained remission after IFX treatment had higher concentrations of amino acids and butyrate in fecal samples [87]. These alterations in feces may be potential prognostic biomarkers in pediatric CD patients.

Fecal microbiota may also predict the efficacy of anti-TNF antibody [i.e. adalimumab (ADA)] in CD. Increased protective microbiota (such as *Barnesiella, Anaerostipes, Tyzzerella, Lachnoclostridium*) and decreased pathogenic bacteria (*E. coli-Shigella*) were observed in ADA-responsive fecal samples. Moreover, the gene *bgIX*, encoding b-glucosidase, and the gene *gph*, encoding phosphoglycolate phosphatase, were also enriched in fecal samples from the ADA-responsive group. However, the abundance of genes encoding ATP-binding cassette was significantly increased in fecal samples from the ADA non-responsive group [88].

Alteration in the intestinal microbiota is observed in UC patients in response to treatment with steroids and biological agents, indicating the potential for gut microbiota profiling to predict UC treatment outcomes. Responders to steroid therapy exhibit changes in β-diversity and enrichment of beneficial bacteria, including *Blautia, Anaerostipes*, and *Bifidobacterium*, compared to non-responders [89]. In UC patients receiving anti-α4β7 monoclonal antibody (mAb) (i.e. vedolizumab), responders at 14 weeks show increased abundances of an unannotated genus from the *Barnesiellaceae* family, whereas non-responders at 14 weeks are more associated with *Collinsella*. Moreover, the genus *cc_115* from the *Erysipelotrichaceae* family was more prevalent in non-responders at 52 weeks [90].

Taken together, these approaches have revealed that microbial composition and metabolic activity are significantly correlated with therapeutic outcomes, paving the way for personalized treatment strategies based on an individual's microbiota profile.

### Adjustment of the intestinal microbiota is used in the clinical treatment of IBD

Modulation of the gut microbiota presents a promising therapeutic avenue in treating IBD, as reflected in the beneficial effects of probiotics. Commonly used strains in the probiotic industry, including *Lactobacillus, Bifidobacterium, Lactococcus, Streptococcus thermophilus, E. coli* Nissle 1917, and *Saccharomyces boulardii*, have shown potential in maintaining gut health. Specifically, *Bacillus coagulans* BC198 has demonstrated its ability to fortify intestinal barrier functions, mitigate inflammatory cell infiltration in the colon, and rebalance the gut microbiota, marking it as a viable probiotic candidate for the management of IBD [91]. The efficacy of probiotics is attributed to their multifaceted roles in immune modulation, stress resistance, pathogen inhibition, microbiota adjustment, and bolstering the intestinal epithelial barrier, making them effective in both inducing and maintaining remission in IBD. Probiotics have been used in many intestinal disorders, such as irritable bowel syndrome [92], and have also shown promise in the treatment of IBD patients [93]. The utilization of probiotics, prebiotics, and synbiotics has been demonstrated to be effective in inducing and maintaining remission in IBD and decreasing the UC disease activity index [94]. Intestinal microbiota-derived extracellular vesicles are thought to play a key role in bacteria–host communication [95], and extracellular vesicles derived from probiotic *Clostridium butyricum* have been proven to protect the intestinal barrier function in UC [96]. In addition, probiotic agents, such as paraprobiotics (inactivated bacteria or their components) and postprobiotics (bacterial metabolites or equivalent synthetic products), are easier to store and manufacture, and cause fewer side effects, making them a potential adjunctive treatment modality [97].

Innovations in microbial engineering have expanded the therapeutic potential of microorganisms [98]. For instance, genetically modifying *Saccharomyces cerevisiae* to produce butyrate has resulted in increased intestinal levels of this beneficial metabolite, offering promise in both clinical and preclinical settings [99]. Furthermore, artificial-enzyme-modified *B. longum* has been developed to enhance antioxidant activity within the gut, showcasing the ability to modulate the immune system and reduce inflammation in IBD models [100]. In another study, an oral probiotic *E. coli* Nissle 1917 was genetically engineered to overexpress catalase and superoxide dismutase for the treatment of intestinal inflammation [101].

FMT represents another frontier in IBD treatment. By transferring fecal microbiota from healthy donors to patients, FMT aims at restoring a healthy gut microbial balance, thus offering a novel therapeutic strategy that has already proven successful in treating recurrent *Clostridium difficile* infections with high cure rates [7]. Several lines of evidence have demonstrated the efficacy of FMT in the treatment of IBD, especially UC, inducing significant remissions and improving the intestinal microbial diversity of patients. A recent study has found that its preparation method may be related to treatment outcomes, e.g. frozen FMT samples or washed microbiota transplantation may have better efficacy or safety [102], which requires further investigation. As a new treatment modality, more randomized, double-blind clinical trials should be conducted in the future to establish clear indications and treatment options for FMT in IBD. The potential of FMT in IBD treatment lies in its capacity to modify the gut ecosystem favorably, demonstrating the critical role of host–microbiota interactions in managing the disease and achieving remission.

Nutritional intervention can regulate the intestinal microbiota and is a common adjuvant treatment strategy for IBD [103]. Several nutritional interventions, including enteral nutrition and dietary control, significantly affect the intestinal microbiota, thus promoting clinical remission and mucosal healing [104]. Notably, other dietary interventions, including a specific carbohydrate diet, a mediterranean diet, and a low-fat diet, also affect the intestinal microbiota, disease activity, and gastrointestinal symptoms in IBD [105]. These emerging therapeutic approaches underscore the significant impact of gut microbiota modulation on treatment strategies for IBD, offering a new horizon for patient care through the restoration of microbial balance and function.

## Conclusions

The intestinal microbiota plays an important role in the physiological regulation of intestinal mucosal homeostasis, and alterations in the intestinal microbiota are involved in the pathogenesis of IBD. Study of the intestinal microbiota and metabolites helps us elucidate the pathogenesis of IBD and has the potential to be applied in the precision diagnosis, prognosis, and drug efficacy evaluation of IBD. By adjusting the intestinal microbiota, selecting specific microorganisms, or FMT, the intestinal flora can be used in the clinical treatment of IBD.

Despite remarkable progress in the field of IBD microbiology, many issues remain to be resolved before precision microbial diagnosis and treatment can be achieved. Evidence for microbial therapies such as probiotics and FMT is insufficient, so more prospective studies are warranted in the future, along with further discovery of microbial predictors to identify appropriate treatment populations. It is also necessary to combine multi-omics analysis to develop precision and personalized microbial medicine for IBD. In addition, if microbial therapy is to be attempted in clinical application in the future, it is essential to optimize the composition, dosage form, and administration regimen of microbial agents. While the intestinal microbiota is a promising diagnostic and therapeutic target for IBD, its clinical application remains a long-term goal.

## Abbreviations

IBD: inflammatory bowel disease; CD: Crohn's disease; UC: ulcerative colitis; SCFA: short-chain fatty acids; Tregs: regulatory T cells; SFB: *segmented filamentous bacteria*; Th: helper T cells; FMT: fecal microbiota transplantation; GPR: G protein-coupled receptor; IFX, infliximab; MAM: microbial anti-inflammatory molecule; MAT: mesenteric adipose tissue; ADA: adalimumab.
